# Female dental students’ perceptions of patient safety culture: a cross sectional study at a middle eastern setting

**DOI:** 10.1186/s12909-018-1415-8

**Published:** 2018-12-10

**Authors:** Khaled Al-Surimi, Haya AlAyadi, Mahmoud Salam

**Affiliations:** 1 Department of Health Systems Management, College of Public Health and Health Informatics, King Abdullah International Medical Research Center, King Saud bin Abdulaziz University for Health SciencesMinistry of the National Guard Health Affairs, Riyadh, Saudi Arabia; 2 Primary Care and Public Health Department, School of Public Health, Imperial College, London, UK; 3 Division of Population and Patient Health, King’s College London Dental Institute at Guy’s, King’s College and St. Thomas Hospitals, London, UK; 4 Department of Community Dentistry, King Saud bin Abdulaziz University, Riyadh, Saudi Arabia; 5 Science and Technology Unit, King Abdullah International Medical Research Center, King Saud bin Abdulaziz University for Health Sciences, King Abdulaziz Medical City, Ministry of National Guard – Health Affairs, PO 22490, (Mail Code 1515), Riyadh, 11426 Saudi Arabia

**Keywords:** Perception, Patient safety, Culture, Dental, Students, Middle East

## Abstract

**Background:**

Patient safety is an integral part of all health care specialties, including dentistry. Dental students are exposed to patient safety culture during their clinical training. The aim of this study was to evaluate the perception of female students enrolled in dental degrees and dental hygiene programs towards patient safety culture and to determine its associated factors at a Middle Eastern setting.

**Methods:**

This is a cross sectional study, based on a self-administered, English language questionnaire distributed by convenience among female dental students enrolled in two major Colleges of dentistry in Riyadh, Saudi Arabia. Participants had fulfilled at least one year of clinical training. Sample characteristics included the specialty and years of clinical training. Student’s perception was measured using the validated Safety Attitude Questionnaire (SAQ) that consists of 36 statements, distributed over six domains. Responses were rated on a five point Likert scale and the average positive response rate (APRR) was calculated. Binary logistic regression models were constructed to determine factors significantly associated with positive perceptions.

**Results:**

The response rate of both student programs was 221/312(70.8%). Students of dental sciences and dental hygiene programs were 133(60.2%) and 88(39.8%) respectively. Almost 42% of students were in their 1st and 2nd years of clinical training. The APRR of: Team Work Climate domain was 54.4 ± 28.0, Safety Climate domain was 51.4 ± 29.7, Job Satisfaction domain was 64.5 ± 33.8, Stress Recognition domain was 56.2 ± 37.8, Perceived Management Support domain was 50.7 ± 37.7, and Working conditions was 55.3 ± 32.1. Female students in their 3rd and 4th year of clinical were adj.OR = 2.3[1.3–4.0] times more likely to have positive perception regarding the team work climate domain when compared to 1st and 2nd year clinical students, *P* = 0.005. At each of the six individual domains, the odds of having a positive perception were also significantly higher among dentistry students in comparison to dental hygiene students with a range of adj.OR 2.6–4.6.

**Conclusions:**

Apparently patient safety is a concern among female dental students enrolled in dental degree and dental hygiene programs. This requires more attention from the staff, dental college's leadership/management, and faculty/students. Perception of dental students towards patient safety culture is expected to improve with the increase of clinical training.

**Electronic supplementary material:**

The online version of this article (10.1186/s12909-018-1415-8) contains supplementary material, which is available to authorized users.

## Key points

- Patient safety is an integral part of all healthcare specialties, including dentistry.
- Dental academicians and students should establish the foundations of a high quality culture of patient safety early in their training before graduation.
- Female dental students, especially dental hygiene students, are evidently concerned about patient safety.
- Perceptions of dental students towards the culture of patient safety are expected to improve while advancing in their clinical experience.
- Integrating patient safety culture within the dental curricula is necessary to assure patient safety and high quality of care in the dental care services.

## Background

Patient safety is an integral part of any healthcare quality improvement system and the foundation of all other components of quality care [1, 2]. A 2007 Institute of Medicine (IOM) report stated that there should be a focus on the conceptual components of quality, not just the indicators to be measured. These components include safe, effective provision of healthcare that is patient-centered, timely, efficient, and equitable [2]. From evidence-based research, the World Health Organization (WHO) noted that at least 10% of patients in developed countries have been injured because of an unsafe medical practice [3]. Comprehensive, equivalent data from developing and/or transitional economy countries is limited, but it is speculated that 2.5–18.4% of all admitted patients are subject to at least one type of adverse event [4]. WHO defined patient safety as the prevention of errors and adverse effects to patients associated with health care. Patient safety, and injuries caused by the healthcare sector, is of such great concern that WHO has established the World Alliance for Patient Safety initiative to address this problem globally [3]. In the Middle East, national and local data about patient safety is scarce; this warrants further research to determine the causes and extent of unsafe care [3].

The IOM has recommended that organizations address the culture of patient safety in their workplace [5]. A culture of patient safety is a system of care delivery that prevents harm to patients, built on safety that involves health care professionals, organizations and patients [6]. It is the product of individual and group values, attitudes, perceptions, competencies, and patterns of behavior [7]. For healthcare students and early career healthcare professionals, it is mandatory to understand and demonstrate appropriate knowledge and skills in the area of patient safety [8]. Most health and medical education curricula focus primarily on competency in medical knowledge, technical skills and judgment [9]. Few health faculties have introduced patient safety-specific teaching into their programs; others have included it as a core component of the curriculum. The WHO Patient Safety Curriculum Guide for Medical Schools, issued in 2009, concentrated on 11 topics based on the Australian Patient Safety Education Framework [10]. Research findings suggested that by introducing patient safety topics into the academic curriculum, students at colleges of health sciences are exposed to better practice, thus become safer healthcare practitioners of the future [8, 11].

There is an imperative need to assess students’ perceptions about the principles of patient safety. Despite its importance, few dental schools (i.e. dental sciences and dental hygiene faculties) have incorporated topics about patient safety in their academic curricula [8, 12, 13]. This may be because morbidity, mortality and their financial impact in dental clinics is low compared to other healthcare settings [14]. One study described the development and evaluation of a three-day patient safety curriculum to advance knowledge, self-efficacy and system-based thinking among dental students; a model previously implemented by Johns Hopkins School of Medicine in the USA. After completing the course, students showed significant improvements in self-efficacy and safety skills [15].

The culture of patient safety is affected by several factors such as the organizational aspects (safety climate and morale), work environment factors (staffing levels and managerial support), team factors (teamwork and supervision), and staff factors (overconfidence and being overly self-assured), that all should be assessed comprehensively [7]. The establishment and maintenance of a culture of patient safety has seldom been studied among the population of female dentists in Middle Eastern countries such as Saudi Arabia. In Saudi Arabia, university faculties are segregated by gender. The first college of dentistry in Saudi Arabia was established in 1975, but admission was restricted to male students until 1978 [16]. The increasing population in Saudi Arabia has led to a demand for more dental clinics, yet not all dental colleges in this country enrol female dental students. As of December 2017, the number of licensed dentists (specialized and non-specialized) in Saudi Arabia, (of all nationalities) was 93,966, with only 25,249 (26.9%) being of Saudi origin [17]. Female Saudi dentists comprised only 1,438 (8.5%) of the dental workforce [18]. Therefore, the aim of this study was to evaluate the perceptions of female dental degrees and dental hygiene students about the culture of patient safety and its associated factors in a Middle Eastern setting.

## Methods

This cross-sectional study was based on a self-administered survey conducted at King Saud bin Abdulaziz University-College of Dentistry and Riyadh Private Dental College between November 2016 and January 2017. The study was approved by the Institutional Review Board of the Saudi Ministry of National Guard – Health Affairs (protocol number RC13/036), and permission was received from the administrators of both colleges.

Using a convenience sampling method, an envelope containing a cover letter, written informed consent form and English language questionnaire was distributed in person to all students of dental sciences and dental hygiene programs enrolled in the targeted settings. An anticipated sample size of 277 was calculated, based on an assumed response rate of 50% favorable positive perceptions, a 95% confidence level and a 6% margin of error.

Participants were included if they were Saudi, female undergraduate students of dental sciences (six year program and one year internship) or dental hygiene (four year program), who had completed at least one full year of training in dental clinics (working on real patients) and supervised by mentors. Students of dental sciences start their clinical training at their 4rth academic year, while dental hygiene program students start their clinical training at their 2^nd^ academic year. Students who had not started working on real patients (e.g., those using manikin simulation training) were excluded. This ensured that study participants had experienced real working environments and would therefore be able to report their perceptions towards the culture of patient safety within those environments.

The cover letter explained the study objectives and informed students that their participation was voluntary. Students of both programs were assured that the data collected would be used only for research purposes, thus maintaining their privacy and the confidentiality of their feedback. The informed consent form stated participants’ right to withdraw from the study without constraint at any time. Study participants responded within one to two days after receiving the survey. Students who failed to return the distributed surveys beyond the second day were not reminded to fill them out in order to avoid participation bias, so they were dropped out.

The questionnaire included questions about students’ characteristics (age, type of college, specialty, and years of clinical training). Clinical training was categorized into two groups. Group one were students of dental sciences and dental hygiene program who both fulfilled their 1^st^ and 2^nd^ year of clinical training versus group two who were dental science students who fulfilled their 3^rd^ and 4^th^ year of clinical training with dental hygiene students who fulfilled their 3^rd^ year). The outcome characteristics were measured using the Safety Attitude Questionnaire (SAQ) that was developed by the Foundation and Agency for Healthcare Research and Quality [7, 19, 20]. It was designed to assess six domains of safety climate: ‘Teamwork Climate’ (six statements), ‘Job Safety Climate’ (eight statements), ‘Job Satisfaction’ (five statements), ‘Stress Recognition’ (four statements), ‘Perceptions of Management’ (six statements), and ‘Working Conditions’ (seven statements). Responses were rated on a five-point Likert scale (‘strongly disagree’, ‘disagree’, ‘neutral’, ‘agree’, ‘strongly agree’). The original version of the SAQ was customized to be more appropriate for dental students. Minor modifications included using the terms ‘dental clinical area’ instead of ‘hospital clinical area’, and ‘student’ instead of ‘nurse’. The modified version of SAQ was piloted among a sample of 23 students from both colleges. Internal consistency testing yielded Cronbach’s alpha values between 0.6 and 0.81 across the six domains (Additional file 1).

SPSS version 25 (IBM, NY, USA) was used for data analysis. Descriptive statistics of categorical variables such as student characteristics were presented as frequencies and percentages. For each SAQ statement, ‘strongly disagree’ and ‘disagree’ responses were grouped as a ‘negative’ response, while ‘agree’ and ‘strongly agree’ answers were grouped as ‘positive’ responses. These were presented as frequencies and percentages.

To calculate the average positive response rate (APRR), individual SAQ statements were rated as 0 for those who responded with ‘strongly disagree’, ‘disagree’ or ‘neutral’, and as 1 for those who responded with ‘agree’ or ‘strongly agree’. APRR was then calculated by dividing the number of positive perception statements by the total number of statements per domain. The APRR of all students across individual domains was then presented as mean ± SD. Adjusted by the academic specialty, students with mean APRR scores of above 50 (above neutral response) for any specific domain were counted as having a positively perceived domain, while those with scores of 50 and below were counted as having a negatively perceived domain [21]. However, some studies such as Nordén-Hägg et al, adopted the cut-off point of percentage mean score ≥75 as indicator of positive responses to SAQ statements [22]. The overall score of SAQ domains was neither investigated in this study nor in previous literature, as the value of this tool is in its ability to assess and recommend improvements in the safety culture at its six specific targets or domains [23].

Pearson’s Chi-square test was used to identify higher rates of positive perception across student characteristics. Pearson’s correlation was conducted to describe the relationship between various SAQ domains. A series of binary logistic regression models was constructed to determine factors significantly associated with positive perceptions and the adjusted odds ratio for individual SAQ domains was presented. The level of significance was set after applying the Holm-Bonferroni correction at *P*-value <0.04.

## Results

### Student and outcome characteristics

Of the 312 questionnaires distributed, 221 questionnaires were completed and returned. Response rate was (60.2%, *n*=133) among students of dental sciences, and (39.8%,*n*=88) among students of the dental hygiene program. The mean ± standard deviation of age was comparable in both specialty groups, with average in both 21.9 ± 1.8 years. One hundred and sixty-five students (74.7%) were enrolled at King Saud bin Abdulaziz University (public sector) among whom 97(58.8%) were students of dental sciences. Among the 56 (25.3%) students enrolled in the private Riyadh Dental College, 36(64.3%) were students of dental sciences. The sample contained 92 (41.6%) students in their first or second year of clinical training, and 129 (58.4%) were in their third or fourth year (mean: 2.6 ± 0.9 years), See Table 1. Equal distribution between students of dental sciences and dental hygiene was observed throughout the clinical training years.

**Table 1:** Sample and outcome characteristics

	*N* = 221(70.8%)
Age (mean ± SD)	21.9 ± 1.8
Type of college	
Private Public	56(25.3) 165(74.7)
Specialty	
Dental sciences Dental hygiene	133(60.2) 88(39.8)
Years of clinical training	
One Two Third Four Mean ± SD	36(16.3) 56(25.3) 85(38.5) 44(19.9) 2.6 ± 0.9

*n* frequency, %, percentage, *SD* standard deviation

Responses to individual SAQ statements are presented in Table 2. For the ‘Teamwork Climate’ domain, the APRR was 54.4 ± 28.0, with the highest positive response observed when the students were asked whether or not “it is easy to ask questions if something is not understandable”. In the ‘Safety Climate’ domain, the APRR was 51.4 ± 29.7, and the highest positive response was observed when the students were asked whether they “received appropriate feedback about their performance” (134; 60.6%). For the ‘Job Satisfaction’ domain, the APRR was 64.5 ± 33.8, and the highest positive response was noted in response to the statement asking whether the student “liked her specialty” (167; 75.5%). In the ‘Stress Recognition’ domain, the APRR was 56.2 ± 37.8, and the highest positive response was to the statement, “I am less effective when I feel fatigued” (134; 60.6%). In terms of the ‘Perceived Management Support’ domain, the APRR was 50.7 ± 37.7, and the highest positive response was to whether “the clinical supervisor was doing a good job” (134; 60.6%). Finally, the APRR of the ‘Working Conditions’ domain was 55.3 ± 32.1, with 138 (62.4%) positively responding that “students had good collaborations with their colleagues”. Analysis of the interrelationships between the six SAQ domains revealed that all were positively and significantly correlated with each other (Table 3).

**Table 2:** Responses to the Safety Attitude statements

	Negative *n*(%)	Neutral *n*(%)	Positive *n*(%)
Team work climate **(**Cronbach’s α = 0.60)			
1. Students input is well received in this clinical area.	18(8.0)	98(44.5)	107(48.5)
2. In this dental clinic, it is difficult to speak up if I perceive a problem with patient care.	84(38.0)	63(28.5)	74(33.5)
3. Disagreements in this clinical area are resolved appropriately (i.e., not who is right, but what is best for the patient).	33(14.9)	88(39.8)	100(45.3)
4. I have the support I need from other personnel to care for patients.	18(8.1)	61(27.6)	142(64.3)
5. It is easy for students here to ask questions when there is something that they do not understand.	25(11.4)	40(18.1)	156(70.5)
6. The supervisors and students here work together as a well-coordinated team.	29(13.1)	58(26.2)	103(60.7)
Average positive response rate (Mean ± SD)	54.4 ± 28.0		
Safety climate **(**Cronbach’s α = 0.75)			
7. I would feel safe being treated here as a patient.	45(20.4)	75(33.9)	101(50.7)
8. Medical errors are handled appropriately in this clinical area.	20(9.1)	76(34.4)	125(56.5)
9. I know the proper channels to direct questions regarding patient safety in this clinical area	23(10.4)	81(36.7)	117(52.9)
10. I receive appropriate feedback about my performance.	25(11.3)	62(28.1)	134(60.6)
11. In this dental clinic, it is difficult to discuss errors.	85(38.5)	67(30.3)	69(31.2)
12. I am encouraged by my colleagues to report any patient safety concerns I may have	11(5.0)	81(36.7)	129(58.3)
13. The environment in this dental clinic makes it easy to learn from the errors of others.	30(13.6)	67(30.3)	124(56.1)
14. My suggestions about safety would be acted upon if I expressed them to management.	27(12.2)	101(45.8)	93(42.0)
Average positive response rate (Mean ± SD)	51.4 ± 29.7		
Job satisfaction (Cronbach’s α = 0.76)			
15. I like my specialty	19(8.7)	35(15.8)	167(75.5)
16. Practicing here is like being part of a large family.	24(10.9)	56(25.3)	141(63.8)
17. This is a good place to practice.	35(15.8)	55(24.9)	131(59.3)
18. I am proud to practice in this dental clinic	34(15.4)	45(20.4)	142(64.2)
19. Ethics in this dental clinic is high.	8(9.5)	68(30.8)	132(59.7)
Average positive response rate (Mean ± SD)	64.5 ± 33.8		
Stress recognition (Cronbach’s α = 0.76)			
20. When my workload becomes excessive, my performance is impaired.	25(11.4)	77(34.8)	119(53.8)
21. I am less effective at work when fatigued.	19(8.6)	68(30.8)	134(60.6)
22. I am more likely to make errors in tense or hostile situations	32(14.5)	58(26.2)	131(59.3)
23. Fatigue impairs my performance during emergency situations.	41(18.6)	67(30.3)	113(51.1)
Average positive response rate (Mean ± SD)	56.2 ± 37.8		
Perceived management support (Cronbach’s α = 0.81)			
24. Clinical management supports my daily efforts	15(6.8)	98(44.3)	108(48.9)
25. Clinical management doesn’t knowingly compromise patient safety	30(13.6)	89(40.3)	102(46.1)
26. Clinical supervisor is doing a good job	31(14.5)	55(24.9)	134(60.6)
27. Problem personnel are dealt with constructively by our clinical units	21(9.5)	97(43.9)	103(46.6)
28. I get adequate, timely info about events that might affect my work	35(15.9)	78(35.3)	108(48.8)
29. The levels of students in this dental clinic are sufficient to handle the number of patients.	37(16.7)	67(30.3)	117(53.0)
Average positive response rate (Mean ± SD)	50.7 ± 37.7		
Working conditions (Cronbach’s α = 0.77)			
30. This dental clinic does a good job of training new personnel.(e.g. students or staff)	29(23.1)	60(27.1)	132(59.8)
31. All necessary information for diagnostic and therapeutic decisions is routinely available to me.	16(7.3)	73(33.0)	132(59.7)
32. Trainees in my discipline are adequately supervised.	6(9.5)	68(30.8)	132(59.7)
33. I experience good collaboration with students in this clinical area.	13(5.9)	70(31.7)	138(62.4)
34. I experience good collaboration with dental staff in this dental clinic.	24(10.8)	73(33.0)	124(56.2)
35. I experience good collaboration with booking staff in this dental clinic.	62(28.1)	74(33.5)	85(38.4)
36. Communication barriers that lead to delays in delivery of care are common.	112(50.6)	76(34.5)	33(14.9)
Average positive response rate (Mean ± SD)	55.3 ± 32.1		

*n* frequency, % percentage

**Table 3:** Pearson’s correlation between the average positive response rates of various SAQ domains

	Team work climate	Safety climate	Job satisfaction	Stress recognition	Management support
Safety climate	0.647				
	*P* < 0.001*				
Job satisfaction	0.569	0.666			
	*P* < 0.001*	*P* < 0.001*			
Stress recognition	0.167	0.326	0.371		
	*P* = 0.013*	*P* < 0.001*	*P* < 0.001*		
Perceived management support	0.550	0.714	0.614	0.345	
	*P* < 0.001*	*P* < 0.001*	*P* < 0.001*	*P* < 0.001*	
Working conditions	0.458	0.670	0.569	0.353	0.714
	*P* < 0.001*	*P* < 0.001*	*P* < 0.001*	*P* < 0.001*	*P* < 0.001*

*P P*-value, * statistically significant at <0.05

### Factors associated with positive perceptions of SAQ domains

From the sample of female dental students enrolled in both specialties in our study, there were significantly more negative perceptions within the domain of ‘Safety Climate’ (131; 59.3%) than those with positive perceptions (90; 40.7%, *P* = 0.006). Significantly more students had positive perceptions within the ‘Job Satisfaction’ (145, 65.6%) and ‘Working Condition’ domains (126, 57%) than negative perceptions (*P* < 0.001 and *P* = 0.037, respectively). Students in their third or fourth years of clinical training (75, 58.1%) had significantly more positive perceptions within the ‘Teamwork Climate’ domain than students in their first or second years (33, 35.9%, *P* = 0.001). There were no statistically significant differences between responses to statements in any domain in terms of type of college.

The highest positive response rate among students of dental degree was observed at job satisfaction (78.9%) and working conditions (66.2%) domains which were significantly higher than students of dental hygiene, (45.5%, *P*<0.001) and (43.2%, *P*=0.001) respectively. The least positive response rates were observed among students of dental hygiene at the safety climate (25.0%) and perceived management support (28.4%), compared to students of dental sciences who had higher positive response rates, (51.1%, *P*<0.001) and (55.6%, P<0.001). All in all, women studying on academic dental specialty had significantly more positive perceptions across all six domains compared with dental hygiene students (Table 4).

**Table 4:** Positive perception rates of various SAQ domains across students' characteristics

	*N*(%)	Clinical training		Type of college		Specialty	
	221(100)	1–2	3–4	Private	Public	Dentistry	Hygienist
Team work climate							
Positive Negative	108(48.9) 113(51.1)	33(35.9) 59(64.1)	75(58.1) 54(41.9)	26(46.4) 30(53.6)	82(49.7) 83(50.3)	80(60.2) 53(39.8)	28(31.8) 60(68.2)
	*Χ*^*2*^ *= 0.113, P = 0.737*	*Χ*^*2*^ = 10.66, P = 0.001*		*Χ*^*2*^ *= 0.179, P = 0.672*		*Χ*^*2*^ = 17.013, P < 0.001*	
Safety climate							
Positive Negative	90(40.7) 131(59.3)	35(38.0) 57(62.0)	55(42.6) 74(57.4)	28(50.0) 28(50.0)	62(37.6) 103(62.4)	68(51.1) 65(48.9)	22(25.0) 66(75.0)
	*Χ*^*2*^ = 7.606,P = 0.006*	*Χ*^*2*^ *= 0.469, P = 0.493*		*Χ*^*2*^ *= 2.674, P = 0.102*		*Χ*^*2*^ = 14.977, P < 0.001*	
Job satisfaction							
Positive Negative	145(65.6) 76(34.4)	58(63.0) 34(37.0)	87(67.4) 42(32.6)	107(64.8) 58(35.2)	38(67.9) 18(32.1)	105(78.9) 28(21.1)	40(45.5) 48(54.5)
	*Χ*^*2*^ = 21.543,P < 0.001*	*Χ*^*2*^ *= 0.460, P = 0.497*		*Χ*^*2*^ *= 0.168, P = 0.682*		*Χ*^*2*^ = 26.33, P < 0.001*	
Stress recognition							
Positive Negative	110(49.8) 111(50.2)	50(54.3) 42(45.7)	60(46.5) 69(53.5)	34(60.7) 22(39.3)	76(46.1) 89(53.9)	84(63.2) 49(36.8)	26(29.5) 62(70.5)
	*Χ*^*2*^ *= 0.005,P = 0.946*	*Χ*^*2*^ *= 1.319, P = 0.251*		*Χ*^*2*^ *= 3.591, P = 0.058*		*Χ*^*2*^ = 23.934, P < 0.001*	
Perceived management support							
Positive Negative	99(44.8) 122(55.2)	41(44.6) 51(55.4)	58(45.0) 71(55.0)	29(51.8) 27(48.2)	70(42.4) 95(57.6)	74(55.6) 59(44.4)	25(28.4) 63(71.6)
	*Χ*^*2*^ *= 2.394,P = 0.122*	*Χ*^*2*^ *= 0.003, P = 0.953*		*Χ*^*2*^ *= 1.48, P = 0.224*		*Χ*^*2*^ = 15.878, P < 0.001*	
Working conditions							
Positive Negative	126(57.0) 95(43.0)	52(56.5) 40(43.5)	74(57.4) 55(42.6)	35(62.5) 21(37.5)	91(55.2) 74(44.8)	88(66.2) 45(33.8)	38(43.2) 50(56.8)
	*Χ*^*2*^ = 4.348,P = 0.037*	*Χ*^*2*^ *= 0.016, P = 0.901*		*Χ*^*2*^ *= 0.921, P = 0.337*		*Χ*^*2*^ = 11.415, P = 0.001*	

*n* frequency, % percentage, χ^2^ Pearson Chi-square, *P P*-value, * significant at *P* < 0.04

A series of logistic regression analyses were constructed to adjust for any possible confounding effect between the years of clinical training and dental specialty. Female students in their third or fourth years of clinical training were 2.3 (CI: 1.3–4.0) times more likely to have a positive perception of the ‘Teamwork Climate’ than junior students (adjusted *P* = 0.005). For each of the six individual domains, students of dental sciences were significantly more likely to have positive perceptions compared with dental hygiene students (range of adjusted OR: 2.6–4.6) (Table 5).

**Table 5:** Factors associated with the positive responses of SAQ domains

	Clinical training (years) 1–2^0^; 3–4^1^	Specialty Hygiene^0^; Dentistry^1^
	adj.OR [95% CI]	adj.OR [95% CI]
Team work climate	2.3[1.3–4.0]	3.0[1.7–5.4]
	Adj.*P* = 0.005*	Adj.*P* < 0.001*
Safety climate	1.0[0.6–1.9]	3.1[1.7–5.7]
	Adj.*P* = 0.875	Adj.*P* < 0.001*
Job satisfaction	1.0[0.5–1.8]	4.5[2.5–8.2]
	Adj.*P* = 0.974	Adj.P < 0.001*
Stress recognition	0.6[0.3–1.0]	4.6[2.5–8.3]
	Adj.*P* = 0.052	Adj.*P* < 0.001*
Perceived management support	0.86[0.5–1.5]	3.2[1.8–5.8]
	Adj.*P* = 0.606	Adj.*P* < 0.001*
Working conditions	0.9[0.5–1.6]	2.6[1.5–4.6]
	Adj.*P* = 0.723	Adj.*P* = 0.001*

*Statistically significant at *P* < 0.05; ^**0**^**:** reference group, ^1^: compared group, OR: odds ratio, CI: confidence interval; Adj = adjusted

## Discussion

Female dentists’ perception towards the culture of patient safety hasn’t been previously investigated. Two studies have evaluated it among health care workers, with their targeted sample having predominant female gender distribution, 79% and 90% respectively [24, 25]. For instance, the positive response rates of the six dimensions among Chinese health care workers were as follows: Teamwork Climate: 59.31%; Safety Climate: 54.09%; Job Satisfaction: 54.63%; Working Conditions: 46.69%; Recognition of Management: 45.97%; and Stress Recognition: 20.80%, all of which were lower than 60% [24]. Another study conducted among Danish health care workers found that the positive response rates ranged from 42.6% for perception of unit management to 64.8% for teamwork climate [25]. Both studies revealed different results compared to findings in this study, where the range of positive response rate was between 50.7% and 64.7% (lowest being the perceived management support and highest the satisfaction domain). This can be probably attributed to the difference in the scope of practice.

Students of dental sciences and dental hygiene are aware that their profession in future has its share of clinical and psychological risks to the patient, such as wrong tooth extraction, bleeding, allergy, infection and others [26, 27]. Therefore, authors of this study believe that the Safety Attitude Culture questionnaire used in this setting touched upon the students’ perceptions of the culture of patient safety at an early stage within a training environment, at a deeper personal level, and before graduation. It can be hypothesized that the moderately low positive responses to statements in our SAQ questionnaire could be attributed to students’ limited exposure to real life clinical scenarios. It is well known that early clinical experience (field training) helps students develop appropriate attitudes towards their future practice; nevertheless it is important for students to become orientated with the culture of patient safety at early stages of their career [28]. Students might also have had a limited opportunity to associate what has been learned in classes with the reality of the clinical profession [29]. Lesser positive responses might also be attributed to differences in the scope of practice between students of dental sciences and dental hygiene [30]. This implies that patient care and safety protocols differ between the academic courses of both student groups and therefore, their perception will also differ throughout the six domains of patient safety culture.

Preceptors who facilitate communication with their students and maximize teaching time in the clinic actually help their students to approach patient care in a more positive and problem-solving manner [31]. One study reported that ethical disagreements are not common in dentistry, and if they do occur, they are easily resolved [32]. There is no doubt that unresolved conflicts can be stressors, but students tend to be more lenient and accommodating of disagreements than employees. Findings in this study suggest that there might be deficiencies in the interpersonal relationships between students and supervisors, or between students and clinicians as perceived by the students. In clinical professions, the leading causes of errors that compromise patient safety are lack of communication and poor coordination of care between team members [33]. Dental students should acknowledge the fact that patient-centered care is crucial, so clinical training is important in sculpting the students’ future work relationships to creating a safe work climate.

Adverse events caused by errors are rare, yet they are inevitable in any clinical practice, so reporting them is a major patient safety issue [34]. Students of dental sciences and dental hygiene in this study might perceive that committing errors reflects poorly on their personal performance, whereas in fact, there may be other reasons – system-related errors, for example. Besides being a crucial preventive measure, reporting errors to mentors is a good learning opportunity for other students. Therefore, it has been recommended that, during the course of their training, these students should be better prepared for communication and reporting challenges associated with errors in clinical practice [34].

At the student level, satisfaction can be understood as being a measure of the students’ true interest in their pursuit of the dental profession. Authors speculate that students of dental sciences and dental hygiene who are dissatisfied with their future career will be more likely to jeopardize patient safety across all domains of SAQ. In other words, students of both specialties in this setting believed that dissatisfied dentists might seldom report errors which affects patient safety, disturb working relationships with colleagues, increase stress, and are less in productivity. Students of dental hygiene had less positive perceptions in terms of the ‘Job Satisfaction’ domain, probably because of the mismatch in scope of practice, or wages after graduation. In contrast, one study found that students’ motivation to become a dental hygienist was significantly higher than those following an academic route, because they enroll in this program directly after graduating from high school [35].

Students of both specialties in this setting perceived the future workload as a potential impairment of their performance and cause of fatigue. Students of dental sciences, in particular, are more prone to stress because of the academic and financial burdens associated with this type of training, and the effects of continual clinical supervision [36]. However, a positive response on this domain might be attributed to the Hawthorne effect, whereby students might not admit to anything that might affect their grades. Therefore, the responses given to this domain, and probably other domains, might not accurately reflect the students’ real perceptions, and they might change when they take on full-time jobs. Compared with students of dental hygiene, students of dental sciences require more advanced practice and greater responsibility [37], therefore stress is expected to be higher. However, our study found that students of dental sciences had more positive perceptions than students of dental hygiene, indicating that the first group is better at coping with future complex work scenarios.

Some might speculate that it is too early to question students of dental hygiene and dental sciences about their perceptions of managerial support. In a similar previous study, students of dentistry reported highly positive perceptions of ‘organizational learning’, ‘continuous improvement’ and ‘teamwork’ [38]. Although after graduation most dentists work independently at private clinics, this is not the case with dentists employed in healthcare facilities. In this study, there was a lack of awareness on the managerial role in the clinical training setting, because students of both specialties are expected to report directly to their academic supervisors rather than the hospital management. Exposing these students to clinical management at an early stage of their training helps them to refine their interpersonal and communication skills, and facilitates their assimilation into a real-life work environment upon graduation and employment [39].

Students usually have friendly relationships with clinic employees. They are eager to learn and work, and regularly receive support from other members of staff. It was reported that after graduation and employment, the perceptions of the culture of patient safety among graduates of dental professionals’ improves further [38]. Therefore, this stage is critical: students placed in clinical training environments where there is a lack of support often lag behind in terms of skill development, and may even withdraw from education [40].

One study noted a medical–dental discrepancy in students’ perceptions and views towards the culture of patient safety and healthcare quality. This was justified by admitting that there are fundamental differences in the work and workflows of dental clinics versus medical clinics. Another justification was that, in terms of the culture of patient safety, medical students may have had a greater level of maturity than dental students, simply because there was a delay in the incorporation of these recommendations into dental curricula and practice at an earlier stage of career development.

Few limitations were encountered in this study. Associations between perceptions of the culture of patient safety culture, and other factors such as male gender, academic performance, and dental assistants have yet to be tested. Finally, recruiting students enrolled in dentistry colleges from other geographical regions might have boosted the generalizability and representativeness of the sample.

## Conclusions

Female students of dental sciences and dental hygiene are evidently concerned about patient safety, and this requires greater attention by the faculty, especially among dental hygiene students. Perceptions of dental students of both specialties towards the culture of patient safety are expected to improve with increasing years of clinical experience.

### Recommendations

Patient safety is everyone’s responsibility, so both dental academicians and students should establish the foundations of a high quality culture of patient safety early in their training. Determining students’ needs in terms of quality assurance/improvement and patient safety should be followed by efforts to improve their knowledge, understanding and awareness of these matters, thus establishing the foundation to a true culture of patient safety [41]. A considerable level of awareness, knowledge, skills and concern for patient safety is highly recommended to ensure that dental students adopt optimal levels of quality of dental healthcare in future. Dental college faculties and members of management are advised to integrate the principles of patient safety into the academic curricula, similar to what has previously been adopted by medical schools. These principles can be incorporated in national rules, regulations and guidelines, especially for high-risk dental procedures. The number of female students who seek enrollment in dentistry is rising to keep up with market’s demand. Although there are currently no restrictions on female education, further studies are needed to explore the perceived willingness of females to pursue dental education, within the scope of culture of patient safety and history of gender-based restricted education.

## Additional file

Additional file 1: Safety Attitude Questionnaire Survey. The Safety Attitude Questionnaire (SAQ) for dental students. (DOC 114 kb)

## Abbreviations

APRR: Average positive response rate
IOM: Institute of Medicine
OR: Odd Ratio
SAQ: Safety Attitude Questionnaire
WHO: World Health Organization
