# Supplementary material for: Female dental students’ perceptions of patient safety culture: a cross sectional study at a middle eastern setting
Source: BMC Med Educ. 2018 Dec 10;18:301. doi: 10.1186/s12909-018-1415-8 (PMC6288871; doi:10.1186/s12909-018-1415-8)
Supplement: Supplementary file 1 — Safety Attitude Questionnaire Survey. The Safety Attitude Questionnaire (SAQ) for dental students. (DOC 114 kb) [file 12909_2018_1415_MOESM1_ESM.doc]

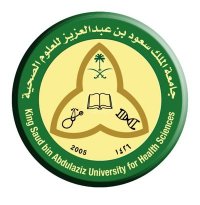
**King Saud bin Abdul Aziz University for Health Sciences**

College of Public Health and Health Informatics

Health System and Quality Management Department

Safety Attitude Questionnaire Survey

Dear participant,

This research aims to assess the culture and attitude of patient safety among the students of dental sciences and dental hygiene. Your participation requires a completion of the survey along with the answering of some demographic questions. Filling this survey will not take more than 15 minutes. Your participation in is voluntary, and you can choose to withdraw from this survey study at any time.

We would like to assure you that your privacy and confidentiality of your answers are highly respected and preserved. The possible benefit of your participation is to raise awareness on patient safety culture and to improve the patient safety education among dental colleges.

By signing this form I acknowledge that I understand the nature of the study, and the means by which my identity will be kept confidential. My signature on this form also indicates that I am 18 years old or older and that I give my permission to voluntarily serve as a participant in the study described.

Signature of Participant___________________ Date _______________

Safety Attitude Questionnaire Survey

| **Birth:** | **Day** | | **Month** | Year | Age:………………….. |
| --- | --- | --- | --- | --- | --- |
| **Gender:** | | | | |  |
| - **Male** - **Female** | | | | |  |
| **You are studying in a :** | | | | |  |
| - **Public University/ college** - **Private University/ college** | | | | |  |
| **What is your specialty:** | | | | |  |
| - **Dentistry** - **Dental Hygiene** | | | | |  |
| **What level or year you are in?** | | Dental students | | | Dental Hygiene |
|  | | - 1^st^ year | | | - 1^st^ year |
|  |  | - 2^nd^ year | | | - 2^nd^ year |
|  |  | - 3^rd^ year | | | - 3^rd^ year |
|  |  | - 4^th^ year | | | - 4^th^ year |
|  |  | - 5^th^ year | | |  |
|  |  | - 6^th^ year | | |  |
|  | | - Internship | | |  |

SECTION 1: **General Information:**

SECTION 2: **Dental Safety Assessment**

| **Safety Attitude Statement** | Strongly  Disagree | Disagree | Neutral | Agree | Strongly  Agree |
| --- | --- | --- | --- | --- | --- |
| **1. Students input is well received in this clinical area.** |  |  |  |  |  |
| **2. In this dental clinic, it is difficult to speak up if I perceive a problem with patient care.** |  |  |  |  |  |
| **3. Disagreements in this clinical area are resolved appropriately (i.e., not who is right, but what is best for the patient).** |  |  |  |  |  |
| **4. I have the support I need from other personnel to care for patients.** |  |  |  |  |  |
| **5. It is easy for students here to ask questions when there is something that they do not understand.** |  |  |  |  |  |
| **6. The supervisors and students here work together as a well-coordinated team.** |  |  |  |  |  |
| **7. I would feel safe being treated here as a patient.** |  |  |  |  |  |
| **8. Medical errors are handled appropriately in this clinical area.** |  |  |  |  |  |
| **9. I know the proper channels to direct questions regarding patient safety in this clinical area** |  |  |  |  |  |
| **10. I receive appropriate feedback about my performance.** |  |  |  |  |  |
| **11. In this dental clinic, it is difficult to discuss errors** |  |  |  |  |  |
| **12. I am encouraged by my colleagues to report any patient safety concerns I may have** |  |  |  |  |  |
| **13. The environment in this dental clinic makes it easy to learn from the errors of others.** |  |  |  |  |  |
| **14. My suggestions about safety would be acted upon if I expressed them to management.** |  |  |  |  |  |
| **15. I like my specialty** |  |  |  |  |  |
| **16. Practicing here is like being part of a large family.** |  |  |  |  |  |
| **17. This is a good place to practice.** |  |  |  |  |  |
| **18. I am proud to practice in this dental clinic** |  |  |  |  |  |
| **19. Ethics in this dental clinic is high.** |  |  |  |  |  |
| **20. When my workload becomes excessive, my performance is impaired.** |  |  |  |  |  |
| **21. I am less effective at work when fatigued.** |  |  |  |  |  |
| **22. I am more likely to make errors in tense or hostile situations** |  |  |  |  |  |
| **23. Fatigue impairs my performance during emergency situations (e.g. emergency resuscitation, seizure).** |  |  |  |  |  |
| **24. Clinical management supports my daily efforts** |  |  |  |  |  |
| **25. Clinical management doesn’t knowingly compromise patient safety** |  |  |  |  |  |
| **26. Clinical supervisor is doing a good job** |  |  |  |  |  |
| **27. Problem personnel are dealt with constructively by our clinical units** |  |  |  |  |  |
| **28. I get adequate, timely info about events that might affect my work** |  |  |  |  |  |
| **29. The levels of students in this dental clinic are sufficient to handle the number of patients.** |  |  |  |  |  |
| **30. This dental clinic does a good job of training new personnel.( e.g. students or staff)** |  |  |  |  |  |
| **31. All the necessary information for diagnostic and therapeutic decisions is routinely available to me.** |  |  |  |  |  |
| **32. Trainees in my discipline are adequately supervised.** |  |  |  |  |  |
| **33. I experience good collaboration with students in this clinical area.** |  |  |  |  |  |
| **34. I experience good collaboration with dental staff in this dental clinic.** |  |  |  |  |  |
| **35. I experience good collaboration with booking staff in this dental clinic.** |  |  |  |  |  |
| **36. Communication barriers that lead to delays in delivery of care are common.** |  |  |  |  |  |

SECTION 3:  **Your Comments**

**Please feel free to write any comments about patient safety, error, or event reporting in your dental education and clinical practice:**

-------------------------------------------------------------------------------------------------------------------------------------------------------------------------------------------------------------------------------------------------------------------------------------------------------------------------------------------------------------------------------------

***THANK YOU FOR COMPLETING THIS SURVEY***
